# Comparison of two nitric oxide supplement formulations on indices of anaerobic power

**DOI:** 10.1186/1550-2783-8-S1-P6

**Published:** 2011-11-07

**Authors:** Allyn Byars, Jason Moriss, Warren Simpson

**Affiliations:** 1 Angelo State University, San Angelo, TX 76909, USA

## Background

Previous studies have indicated that ingestion of nitric oxide (NO) supplements prior to exercise may potentially enhance anaerobic performance. Collectively, NO products are marketed to increase energy, strength, stamina and endurance during anaerobic exercise. However, delivery modes and formula combinations of NO supplements differ in regards to other nutrients included (i.e., creatine, caffeine, etc.) that could possibly impact the efficacy of NO product claims. The purpose of this pilot study was to compare the effects of two NO supplement formulations (NO1 & NO2) on indices of anaerobic power.

## **Methods**

Volunteer subjects included male athletes from a NCAA Division II baseball program (n=6) ages 20-23 years (21.50 +/- 1.05). Subjects performed three 30 second cycle ergometer tests measuring anaerobic power conducted within approximately one week of each other. In this crossover design, each subject ingested the NO1, NO2 or Placebo (PL) in liquid form exactly 30 minutes before each exercise bout. Administration of the trials was double-blinded with the order of the test product ingestion randomized. Peak power (W), average power (W) and fatigue index (% power drop) during anaerobic exercise testing were evaluated.

## **Results**

Using repeated measures ANOVA, results indicated no significant mean differences (p > .05) in peak power between NO1 (827.34 +/- 59.01), NO2 (843.98 +/- 106.49), and PL (761.38 +/- 88.12) trials (p =.215). Mean differences in percent power drop between the NO1 (53.99 +/- 7.01), NO2 (59.91 +/- 3.67), and PL (59.42 +/- 3.84) trials were also not significant (p =.128). Significant mean differences (p ≤.05) in average power existed between the NO1 (548.24 +/- 35.94), NO2 (575.46 +/- 49.13), and PL (547.88 +/- 43.97) trials (p =.005) for the anaerobic cycling protocol used in this study.

## **Conclusion**

Although significant differences in average power were found, peak power and fatigue index were not significantly different between the three anaerobic exercise trials. In addition, practical inferences of the results are limited due to the small sample size. However, the combined results of this investigation may provide meaningful insight. In particular, future studies examining various nutrient combinations used in NO supplements are warranted and may assist coaches and athletes alike regarding ergogenic NO pre-workout options.

